# Nursing students’ immediate responses to distressed clients based on Orlando’s theory

**Published:** 2010

**Authors:** Samereh Abdoli, Shadi Satat Safavi

**Affiliations:** * Nursing, Assistant Professor, School of Nursing and Midwifery, Isfahan University of Medical Sciences, Isfahan, Iran; ** Medical Surgical Nursing, Lecturer, Saveh Branch, Azad Islamic University, Saveh, Iran

**Keywords:** Immediate responses, nursing students, Orlando’s theory, distressed clients

## Abstract

**BACKGROUND:**

Nowadays, problem solving skills, clinical decision making ability and making a proper relationship to clients are essential necessities for nursing graduates; however there are few studies which investigated nursing students’ responses to clients with problematic situations based on nursing theories addressing interactions. The purpose of this study was to analyze the nursing students’ immediate responses to distressed clients’ behaviors focusing on collaborative Orlando’s theory.

**METHODS:**

This exploratory study was assessed 60 nursing students’ responses to a simulated clients’ questionnaire based on Orlando’s theory in 2008. All of the students were enrolled in bachelor degree of nursing. The data were analyzed by dimensional content analysis to specify the key categories, according to concepts of Orlando’s theory.

**RESULTS:**

According to Orlando’s theory, students’ immediate responses to physical and mental problems of distressed clients were classified into 6 main categories: physical caring, uncertainty, assuring, recommending, asking information and explaining. The most frequent responses to clients’ behaviors were physical caring, assuring and recommending and most of the students were unable to respond to mental problems.

**CONCLUSIONS:**

Nursing students responded to clients’ needs automatically and they did not consider clients’ ability in decision making. Medical diagnosis, physical caring and assuring were their main concerns and they were confused in responding to mental problems. Orlando’s theory emphasizes on nurse-client interactions and considers nurses’ perceptions, thoughts, and feelings. It views clients as a participant in care giving, so teaching this theory can enhance students’ communication skills and improve quality of nursing care.

Nowadays, nursing educators are responsible professionally for training graduates who can think critically and solve problems in different clinical settings.^1^–^3^ Problem solving ability is the heart of nursing practice and it needs developing some skills like critical thinking, clinical decision making, making proper diagnosis of problems or in problematic situations, recognizing pattern of clients’ responses, and understanding their feelings and anxieties.^4^–^6^ Nurses are the main members of medical and health organizations and daily they meet clients have their own behaviors and need different decisions and responses in every 30 second moderately.^7^

Clients should be mentioned as participants in care giving and nurses are needed to emphasize on their thoughts, feelings, perceptions and participation in care plans. The clients’ behavior should not be ignored which shows the need for help.^8^ Nurses’ responses based on a correct interaction can improve the feelings of peace and safety in clients.^6^

Nurse-client interaction is the main area in collaborative nursing theories. These theories are focused on improving nurse-client relationship, considering the values in nursing like integrity of human and the necessity of sympathy and human interactions. From their point of view, nursing care is a human process rather than a mechanical practice and it will increase the health and feeling of well being in clients.^9^

Orlando’s theory among collaborative theories is rather special because it generally concentrates on recognizing clients’ emotions and feelings in nursing process. Orlando believes that professional nursing identity is specified only by determining clients’ needs and it could just be validated by clients themselves. Routine and automatic nurses’ responses without considering the meaning of clients’ behaviors just distress the clients and decrease the quality of nursing care.^10^,^11^

Studies have shown that using Orlando’s theory in clinic can improve nurse-clients relationships and decrease distress.^12^,^13^ Orlando’s theory emphasizes on clients’ behaviors (problematic situations), nurses’ immediate responses and nurses’ behaviors (solutions of problems). It believes that problematic situation or client’s behavior in spite of its appearance indicates help request.^14^ Nurse should consider client’s behaviors as needs which have not been met.^15^ In this theory, nurses’ reactions include their perceptions, thoughts and feelings.^16^ Perceptions are interpretation of the client’s behavior and can be considered as the results of the five senses stimulants. Thinking appears as an idea in individual’s mind and is stimulated by perception. Feelings are responses to thoughts and perceptions as well. In other words, they are individual’s subjective expressions according or opposite to perceptions, thoughts or actions.^17^ In Orlando’s opinion, nurse observes the client’s behavior and suggests hypotheses (nurse’s reaction), then these hypotheses or perceptions are altered and validated by client. If the nurse’s action is based on the validated process, the immediate client’s need will be fulfilled and improvement can be achieved.^8^ Indeed Orlando presents a specific collaborative strategy to solve the problematic situations. She believes that nurses should discuss their perceptions, thoughts and feelings with clients verbally and ask them to alter or validate their statements. This process will be continued to result in problem solving and improvement.^16^

The first investigation based on Orlando’s theory related to job problems showed that more than 25% of nurses explained their thoughts instead of feelings. In 58% of cases, head nurses manipulated the problematic situations by themselves and in 50% of cases they dictated their actions to nurses.^15^ Therefore, the immediate nurses’ and head nurses’ responses were automatic.^18^

According to other studies, immediate nurses’ responses to clients’ and their family needs in critical care wards in Hong Kong can be evaluated as positive. This study was not based on collaborative theories, but the results showed that all the main family needs had been met by nurses and physicians.^19^,^20^

On the contrary, the findings of investigating the nurses’ interactions in Iran show that this process has been decreased.^6^,^21^ Sangestani et al in a study on nurse-client interactions in emergency wards suggested that nurses did not have a proper interaction with clients and each communication lasts just 3 minutes moderately.^6^

A suitable and effective human interaction has been one of the essential necessities for nurse graduates so far, but ignoring the client as a care plan participant and presenting routine and automatic nursing care instead of a professional caring have been left as one of the main challenges in nursing education.

Haggerty’s study indicated that nursing students responded to patients’ problems ineffectively and they are more interested in assuring, teaching, recommending and physical care plan. They consider verbal signs more than nonverbal ones and they generally reacted automatically and did not pay any attention to the meaning of clients’ behaviors.^22^

Recent studies have shown that nursing students interacts with clients despotically and mechanically.^23^ Their communications to clients are very short, superficial and based just on physical problems.^5^,^24^ They do not consider interactions and sympathy to clients and they do not have sufficient skills to do it.^25^,^26^

Based on all investigations between 1984 and 1998, interacting with client is one of the main parts of meaningful learning process, learning how to care based on individual, improving the professional development and making competence and self esteem for nursing students.^27^

According to Orlando’s theory, immediate nurses’ responses to problematic situations and distressed clients are essential. Because of limited knowledge about nursing students’ perceptions, thoughts and feelings, results of this research area can provide some information about their behaviors in problematic situations.^10^ and can be considered as a guide for nurse-patient interactions as well. The purpose of this exploratory study was analyzing the immediate nursing students’ responses to distressed clients.

## Methods

By convenient sampling, 95 nursing students were selected from one of the nursing schools in Tehran and just 60 of them agreed to take part in the study. The others dispensed to participate because they did not want to think about questions of the study.

The Haggerty’s questionnaire was used for data collection. This questionnaire is based on Orlando’s nursing process to assess immediate nursing students’ responses to clients who need assistance. The instrument includes two parts: 1- demographic data form requesting students’ sex, age, educational grade and completion of mental health nursing course and 2- simulation of clinical situations about 4 distressed clients. Two first situations regarded to clients with physical problems and the other two situations were about clients with mental problems (Table 1).

**Table 1:** Clinical simulated situations of physical and mental distressed clients

Clinical simulated situation	Verbal behaviors	Nonverbal behaviors
Mr. Karimi is 20 who had surgery of appendectomy without any problem 48 hours ago	“Nurse, I can’t bear my arm pain, it is getting worse, I didn’t get sleep last night”	There are redness of upper site of left arm and problem in IV flow
Mrs Hosseini is 18 who had crash accident. She has two ribs fractured and suffers from chest contusion. She has been admitted from 2 days ago.	“I have a lot of pain during sitting in bed that I can’t move. Bearing this pressure is impossible to me”	She is compressing her chest with her hands. Her body is rigid and her face shows cyanosis.
Mr. Rafiee is 25 who is survived from a very dangerous crash accident and he has been admitted from 2 months ago. Recently satisfying improvements is evident in his situation and he will be discharged next week. His friend died in another similar accident and he is very upset.	“It’s dreadful to be alive while my friend is dead”	He is looking at his friend’s picture and crying
Mrs. Majidi is 22 who bore her first child 3 days ago. She will be discharged tomorrow and there was no problem during delivery.	“Nurse, I still don’t have any feeling to my baby and I’m worried”	She is anxious and she is clenching her fists.

Age, medical diagnosis, physical and mental signs and symptoms and verbal and non-verbal behaviors about each client were presented; then students were asked to write their answers to 4 open questions describing their feelings, perceptions, thoughts and immediate responses based on Orlando’s nursing process. The questions were as follow:

- What did the client say or do which stood out in your mind? (Student’s perception)
- What did you think about what stood out in your mind? (Student’s thought)

What feeling (Emotion) did you expe rience following the thought you indicated above? (Student’s feeling)

4- What would be the first thing you would say to this client if you were their nurse? (Student’s immediate response)

After reading the information about each client, students wrote their reactions and immediate responses in 5 minutes.

To use, translate and cultural alteration of questionnaire the written permission was adopted from its author.

The external and conceptual validity were confirmed by 5 nurse professors. Dimensional content analysis method was used in this study which determines the first codes by theories or previous surveys. Then during the analysis these codes was controlled and refined.^28^ Therefore, students’ responses were written on separate papers in each 4 asked situations. Then these papers were read several times to understand students’ perceptions, thoughts, feelings and immediate responses. The main sentences were extracted and the meaning and concept of each sentence were defined. Afterwards, contents were categorized based on concepts of Orlando’s theory to physical caring, uncertainty, assuring, recommending, asking information and explaining categories.

Skilled coworkers in content analysis were asked to assess data analysis. By asking a variety of participants, generalization of findings were assured.^29^

## Results

Participants’ mean age was 21.7 (20-25 years old); 60% were women and the rest (40%) were men (Table 1).

**Table 1:** Demographic information about students participating in the study

Demographic information	N = 60	
Age	Range	20.5
	Average	21.7
Sex	Female	35
	Male	25
Marital status	Single	47
	Married	13
Educational grade	Third year	38
	Fourth year	22
Completion of mental health nursing course	Yes	60
	No	-
Familiarity to collaborative nursing theories	Yes	-
	No	60

After analyzing, data were categorized to 6 categories including: physical caring, uncertainty, assuring, recommending, asking information, and explaining (Table 2).

**Table 2:** Nursing students’ immediate responses to problematic situations based on Orlando’s theory

Category	Examples
**Physical caring**	I will inject analgesia
	I will prescribe oxygen
	I will change IV line
	Breath in sitting position
	Don’t move your arms

**Asking information**	Talk to me about that
	Talk about your baby

**Recommending**	Calm down/don’t worry
	You should thank God

**Assuring**	It’s normal/no problem
	Everything is going to be ok

**Explaining**	In some people affection to baby needs more time
	You are not responsible for your friend’s death

**Uncertainty**	I don’t know what should I do
	I’m scared, I can’t understand my client’s problem

The results of simulated situation 1 (client suffering from pain) and situation 2 (client suffering from dyspnea) showed that students’ perceptions from verbal and nonverbal clients’ behaviors were based on medical diagnosis more frequently. All of the students felt that their perceptions of client’s problem were correct and they did not show any intention to ask information to validate or alter their thoughts. They assumed that they are always able to solve clients’ problems and none of them experienced uncertainty in immediate responses. In immediate responding to problematic situations, most of the students suggested physical caring like “prescribing oxygen”, “injection of analgesia” or “changing IV line” without explanation. The others showed assuring responses by using some sentences like “this is normal” and “everything is going to be ok”. Some of them recommended to clients to be calm down or thank god without a real understanding of problem. Surprisingly, students considered nonverbal behaviors more than verbal ones.

On the contrary, students could not have a suitable perception in situation 3 (no feeling to newborn baby) and situation 4 (death of a friend). Findings indicated that the students were not only unable to perceive client’s mental distress, but also they labeled them depressed or passionate. They stated that the clients exaggerated the reality. They were uncertain about their diagnoses and encouraged clients to talk about their problems. Indeed asking information occurred in mental situations more than physical situations. All of the students were unable to think about mental problems. Only few students explained that they thought the client was anxious and distressed and they wanted to manage the situation. But they did not know what they should do in such a condition. They expressed uncertainty by using sentences like “I don’t know what I should do” and “I’m scared, I can’t understand the client’s problem”. Others selected assuring and explaining strategies using sentences like “everything will be in order”, “You’re not responsible for your friend’s death” and “to some people affection to baby needs more time”. The other strategy was recommending just being “calm down” or “thanking god”. In opposite to physical problems, students considered to verbal behaviors more than nonverbal ones (Table 3).

**Table 3:** Nursing students’ immediate responses to physical and mental problematic situations based on Orlando’s theory

Category	Responses to physical problematic situations (%)	Responses to mental problematic situations (%)	Total %
Physical caring	68	-	68
Assuring	14	13	27
Asking information	-	9	9
Recommending	8	11	19
Explaining	-	6	6
Uncertainty	-	61	61

## Discussion

The results of this survey indicated that in spite of curriculum emphasizing on nurse-client interactions and clients’ participation in clinical decision making, students did not use their theoretical knowledge. They trusted their hypotheses and perceptions, and did not ask further information from clients and choose just assuring and recommending strategies. They had forgotten their teaching role in all of the situations.

Sangestani et al showed that 56.7% of nurses did not discuss their perceptions with clients, 68.9% of them did not ask clients’ opinions about problems and 81.1% did not ask clients’ ideas about discussing subjects; 71.7% of nurses in their study suddenly interrupted their relationships with clients and 87.8% of them did not introduce themselves and 66.2% did not call clients’ names as well.^6^ But the key point in nursing process, considering individual’s thoughts and client’s situations, is that the nurse’s perceptions about client’s participation are not real and they have to be validated by clients.^18^ Indeed in professional nursing the clients are considered as a whole and medical diagnoses are just a part of data.^30^

This survey showed that students responded to physical problems more effectively and automatically. They were confused in responding to distressed clients with mental problems. Nonjudgmental thinking, which is one of the professional principles, was ignored by labels such as depressed or passionate. Although few students encouraged distressed patients to explain their feelings, but no one suggested sympathetic and supportive nursing implementations such as sitting beside the client, touching their hands and listening to them carefully. They did not concentrate on verbal and non-verbal behaviors simultaneously. Sabzevari et al found that students didn’t choose sympathy and they are weak in nonverbal relationships.^26^

Recent nursing studies based on Orlando’s theory show that students respond to distressed clients’ behaviors are automatically and impatiently and they emphasize just on physical caring.^22^,^23^ These findings about nurse-client interaction process indicate a remaining challenge to nursing students.^25^–^31^ This results state that presenting a nursing care based on client as a whole has been ignored. Facing clients with physical problems is more frequent than mental ones and there is less emphasizing on clinical training about mental and spiritual aspects.

The other point in this study was the dispensing of 35 students because they just did not want to think about this study. This problem should attract the consideration of curriculum designers, nursing society and faculties’ members to pay more attention to improve critical thinking skills in nursing students.

## Conclusion

The nursing education system emphasizes on educating students who will be able to present the best care in future.^5^ But there are some problems in effective and immediate responses to distressed clients in clinics. It seems that nursing curriculums consider teaching the relationship skills more than using interaction process. Therefore, collaborative Orlando’s theory can be used as a clinical guide to specify the nurse role and acquire the interaction skills.^32^ The authors believe that students should be introduced to nursing theories. It is suggested that it is better to teach the relationships process based on nurses’ perceptions, feelings and thoughts.

The authors declare no conflict of interest in this study.
